# On the Age of Information in a Two-User Multiple Access Setup

**DOI:** 10.3390/e24040542

**Published:** 2022-04-12

**Authors:** Mehrdad Salimnejad, Nikolaos Pappas

**Affiliations:** Department of Science and Technology, Linköping University, SE-60174 Norrköping, Sweden

**Keywords:** age of information, multiple access channels, multiple-input multiple-output Rayleigh fading channel, discrete-time Markov chain, convex optimization

## Abstract

This work considers a two-user multiple access channel in which both users have Age of Information (AoI)-oriented traffic with different characteristics. More specifically, the first user has external traffic and cannot control the generation of status updates, and the second user monitors a sensor and transmits status updates to the receiver according to a generate-at-will policy. The receiver is equipped with multiple antennas and the transmitters have single antennas; the channels are subject to Rayleigh fading and path loss. We analyze the average AoI of the first user for a discrete-time first-come-first-served (FCFS) queue, last-come-first-served (LCFS) queue, and queue with packet replacement. We derive the AoI distribution and the average AoI of the second user for a threshold policy. Then, we formulate an optimization problem to minimize the average AoI of the first user for the FCFS and LCFS with preemption queue discipline to maintain the average AoI of the second user below a given level. The constraints of the optimization problem are shown to be convex. It is also shown that the objective function of the problem for the first-come-first-served queue policy is non-convex, and a suboptimal technique is introduced to effectively solve the problem using the algorithms developed for solving a convex optimization problem. Numerical results illustrate the performance of the considered optimization algorithm versus the different parameters of the system. Finally, we discuss how the analytical results of this work can be extended to capture larger setups with more than two users.

## 1. Introduction

Age of Information (AoI) is considered to be a metric for characterizing the timeliness and freshness of data [1,2,3,4]. AoI was first introduced in [4], and it is defined as the time difference between the current time and the time that the latest status update was successfully received by a destination. In [4,5,6,7,8,9], the authors derived the average AoI for systems with different availability of resources using different queuing models. The M/M/1, M/D/1, and D/M/1 queues were studied under the first-come-first served (FCFS) queue management protocols in [4]. In [5,6,7,8,9], the authors considered the last-come-first-served (LCFS) queue protocols with or without the ability to preempt the packet in service. Recently, different types of traffic associated with different source nodes have been considered in which some nodes generate time-sensitive status updates and other nodes strive to achieve high throughput. The performance of a multiple access channel with heterogeneous traffic has been investigated in [10] where one user has bursty arrivals of regular data packets while another AoI-oriented sensor has energy-harvesting capabilities.

The interplay between delay guarantees and information freshness in a two-user multiple access channel with multi-packet reception (MPR) capability at the receiver and heterogeneous traffic is studied in [11]. Motivated by [11], in [12] the interplay of deadline-constrained traffic and the average AoI in a two-user random-access channel with MPR reception capabilities was investigated. The authors obtained analytical expressions for the throughput and drop rate of a user with external bursty traffic, which is the deadline-constrained and analytical expression for the average AoI of a user monitoring the sensor. In [13], the authors presented the analysis of the average AoI with and without packet management at the transmission queue of the source nodes. In the proposed system, each source node has a buffer of infinite capacity to store incoming bursty traffic in the form of packets.

A small average AoI corresponds to having fresh information, which is a key requirement in various applications, including Internet of Things (IoT) scenarios, wireless sensor networks, industrial control, and vehicular networks. The problem of optimizing the process, i.e., of sending status updates from a user to minimize the average AoI, was studied in [14,15,16,17,18,19,20,21,22,23,24]. The works [14,25] consider real-time IoT monitoring systems, where IoT devices sample a physical process and transmit status updates to a remote monitor to minimize the average AoI. In [15], the worst-case average AoI and average peak AoI from a sensor in a system where all other sensors have a saturated queue are analyzed. In [16], a randomized policy, a MaxWeight policy, and a Whittle’s Index policy have been proposed to minimize the AoI subject to minimum throughput requirements. In [17], the problem of minimizing AoI in various continuous-time and discrete-time queuing systems, such as the FCFS G/G/1, the LCFS G/G/1, and the G/G/*∞*, has been studied. In [18], the age-optimal scheduling policies in a network with general interference constraints have been studied. In [19], the authors considered an energy-harvesting sensor and determined the optimal status update policy to minimize the average AoI. In [20], several methods have been proposed for solving an AoI minimizing problem with throughput constraints. In [20,21,22,23,24], the authors developed the Drift-Plus-Penalty (DPP) policy from the Lyapunov optimization theory which is often used for solving stochastic network optimization problems with stability constraints. In [23], the authors applied the Lyapunov DPP method to minimize the average AoI total transmit power of sensors under constraints on the maximum average AoI and the maximum power of each sensor. In [24], the authors proposed the probabilistic random-access (PRA) and DPP methods for solving an optimization problem that aims to minimize the average AoI of the energy-harvesting node subject to the queue stability constraint of the grid-connected node. Recently, the performance of AoI has been investigated in Multiple-Input Multiple-Output (MIMO) systems [26,27,28,29,30]. In [26,27], the user scheduling problem has been investigated to minimize AoI in a multiuser MIMO status update system where multiple single-antenna devices send their information over a common wireless uplink channel to a multiple-antenna access point. In [28], a novel MIMO broadcast setting is studied to minimize the sum average AoI through precoding and transmission scheduling. The age-limited capacity through MIMO setup was investigated in [29], where a random subset of users are active in any transmission period. In [30], the authors analyzed and optimized the performance of AoI in a grant-free random-access system with massive MIMO.

### Contributions

**Figure 1:**
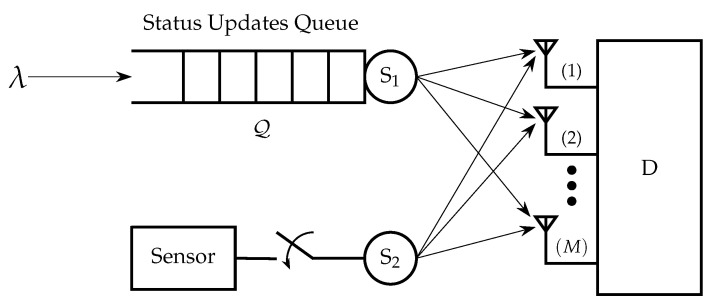
User 1 has AoI-oriented external bursty traffic with probability λ, user 2 has also AoI-oriented traffic but it can control the generation of status updates.

Motivated by [12,24,29] in this paper we consider a multiple access channel (MAC) with two users that have AoI-oriented traffic with different characteristics. The receiver has multiple antennas, and the communication channels are subject to Rayleigh fading and path loss, as depicted in Figure 1. The key contributions of this paper are:

1. We consider a MIMO Rayleigh fading channel in which the receiver has multiple antennas. Most of the literature assumes single-antenna setups.
2. In a two-user multiple access setup, we study the performance of the average AoI of the first user for the cases of FCFS, LCFS with preemption, and queue with packet replacement when external status update packets are arriving according to a Bernoulli process. The case of FCFS can be useful when we do not allow out-of-order transmissions, since we intend to also monitor the evolution of a process in addition to the freshness. The policies of LCFS with preemption and the queue with replacement are more relevant when we do not care for the evolution of the process; thus, we can allow packet dropping.
3. Furthermore, we consider a threshold for the AoI of the second user which affects the sampling and transmission frequency, and we conduct a detailed analysis for the AoI of the second user. In particular, we derive exact analytical expressions for
  - The distribution of the AoI of the second user;
  - The probability that the AoI of the second user is greater than a threshold;
  - The average AoI of the second user;
  - The average AoI of the first user for the LCFS with preemption policy by assuming the threshold for the AoI of the second user.
4. We formulate and analyze a constrained optimization problem where the objective function is the average AoI of the first user for the FCFS queue discipline, with a constraint on the average AoI for the second user, which should be less than a threshold. We show that the problem is not convex in general. Then, we propose a suboptimal approach to solve the problem. Furthermore, we solve the optimization problem where the objective function is the average AoI of the first user for the LCFS scheme with preemption.

The considered setup is expected to occur in several scenarios in wireless industrial automation (Industry 4.0, Industrial IoT), in which several processes are coexisting by sharing the same network resources, and sensing the states of a set of systems is essential.

The remainder of this paper is organized as follows. In Section 2, the system model is introduced. In Section 3, we analyze the average AoI of the first and second users; we formulate an optimization problem and propose a convex optimization algorithm to minimize the average AoI of the first user under the constraint on the average AoI for the second user. In Section 4, we present the numerical and simulation results to evaluate the performance of the proposed optimization method. Conclusions are drawn in Section 6.

## 2. System Model

We consider a time-slotted MAC with two users equipped with a single antenna transmitting their information in the form of packets over a MIMO Rayleigh fading channel to a common receiver with *M* antenna, as shown in Figure 1. We assume that both users have AoI-oriented traffic, but with different characteristics. One of the main differences between the users is that the first one does not have control over the generation of the status update packets, but they are externally generated according to a Bernoulli process with a probability λ, while the second user can control the generation of status update packets. Let Q(t) denote the status update queue of the first user in time slot *t*, which has infinite capacity. When the queue of the first user is not empty, it attempts to transmit its status update packets with a probability $q_{1}$. Additionally, it is assumed that the second user samples and transmits its status updates with probability $q_{2}$ based on a generate-at-will policy. Note that in Section 3.5, we will consider the case where the second user can adjust its sampling and transmission probability based on an AoI threshold.

### 2.1. Physical Layer Model

We assume a quasi-static Rayleigh fading model for the duration of the timeslot in which $h_{i}\in C^{M}$ denotes the M×1 channel vector between the user *i*(i=1,2) and the receiver and reads

(1) $$h_{i}=\sqrt{\beta_{i}}g_{i},$$

where $g_{i}\in C^{M}$ denotes the fast-fading coefficients between user *i* and receiver antenna, and $\beta_{i}$ models path loss where $\beta_{i}=r_{i}^{-\alpha }$. Please note that $r_{i}$ is the distance between user *i* and the receiver and α is the path-loss exponent that 2<α<7. At each time slot, the received signal-to-noise ratio (SNR) at the receiver when only user *i* transmits and the received signal to interference and noise ratio (SINR) at the receiver when both users transmit are given by

(2) $$\begin{array}{cc} {\mathrm{SNR}}_{i} & =\frac{P_{t,i}\beta_{i}{∥g_{i}∥}^{2}}{\sigma^{2}},\\ {\mathrm{SIN}R}_{i} & =\frac{P_{t,i}\beta_{i}{∥g_{i}∥}^{2}}{\sigma^{2}+P_{t,j}\beta_{j}{\left|{\tilde{g_{i}}}^{H}g_{j}\right|}^{2}}, \end{array}$$

where $P_{t,i}$ is the transmitted power by user *i* and $\sigma^{2}$ is the variance of the complex additive white Gaussian noise (AWGN) at the receiver. Please note that $∥g_{i}{∥}^{2}$ follows a gamma distribution with shape parameter *M*, and scale parameter 1 (i.e., $∥g_{i}{∥}^{2}∼\Gamma \left(M,1\right)$). Additionally, it is shown in [31] that ${\tilde{g_{i}}}^{H}g_{j}∼\mathrm{CN}\left(0,1\right)\forall i,j$, where ${\tilde{g_{i}}}^{H}={g_{i}}^{H}/∥g_{i}∥$ and they are mutually independent and independent of $∥g_{i}{∥}^{2}$. In this paper, we assume MPR capability at the receiver, which means that the receiver can correctly decode packets from multiple simultaneous transmissions that are interfering with each other. It is assumed that a packet is successfully transmitted from the user *i* if the received SNR or SINR at the receiver exceeds a certain threshold. The success transmission probability for user *i* when only user *i* transmits $p_{i/i}$ and when both users transmit $p_{i/i,j}$ can be obtained as [31]

(3a) $$\begin{array}{cc} p_{i/i} & =\mathrm{Pr}\left\{{\mathrm{SNR}}_{i}>\gamma \right\}=\int_{\frac{\gamma \sigma^{2}}{P_{t,i}\beta_{i}}}^{\infty }\frac{z^{M-1}e^{-z}}{(M-1)!}dz=\frac{\Gamma \left[M,\frac{\gamma \sigma^{2}}{P_{t,i}\beta_{i}}\right]}{(M-1)!},i=\left\{1,2\right\} \end{array}$$

(3b) $$\begin{array}{cc} p_{i/i,j} & =\mathrm{Pr}\left\{{\mathrm{SIN}R}_{i}>\gamma \right\}=\int_{0}^{\infty }\int_{\left(\frac{\gamma \sigma^{2}}{P_{t,i}\beta_{i}}+\frac{P_{t,j}\beta_{j}}{P_{t,i}\beta_{i}}\gamma t\right)}^{\infty }\frac{z^{M-1}e^{-(z+t)}}{(M-1)!}dzdt,i=\left\{1,2\right\},j\neq i. \end{array}$$

Note that for the special case of M=1, (3a) and (3b) can be written as

(4a) $$\begin{array}{cc} p_{i/i} & =\mathrm{Pr}\left\{{\mathrm{SNR}}_{i}>\gamma \right\}=\exp\left(-\frac{\gamma \sigma^{2}}{P_{t,i}\beta_{i}}\right) \end{array}$$

(4b) $$\begin{array}{cc} p_{i/i,j} & =\mathrm{Pr}\left\{{\mathrm{SIN}R}_{i}>\gamma \right\}=\exp\left(-\frac{\gamma \sigma^{2}}{P_{t,i}\beta_{i}}\right){\left(1+\gamma \frac{P_{t,j}\beta_{j}}{P_{t,i}\beta_{i}}\right)}^{-1},i=\left\{1,2\right\},j\neq i. \end{array}$$

The results presented in this work are general and can also be applied to other types of wireless channels as long as we can calculate the aforementioned success probabilities.

### 2.2. The Service Probability

The service probability of a user is defined as the probability of successful transmission in a timeslot. The service probability for the first user is given by

(5) $$\begin{array}{c} \mu_{1}=q_{1}\left(1-q_{2}\right)p_{1/1}+q_{1}q_{2}p_{1/1,2}. \end{array}$$

To obtain the service probability of the second user, three cases are considered as follows.

1. When the queue of the first user is empty.
2. When the queue of the first user is not empty, and it does not transmit a status update to the receiver.
3. When the queue of the first user is not empty, and it transmits a status update to the receiver with probability $q_{1}$.

The service probability of the second user can be written as

(6) $$\begin{array}{cc} \mu_{2} & =\mathrm{Pr}\left\{Q=0\right\}q_{2}p_{2/2}+\mathrm{Pr}\left\{Q\neq 0\right\}\left(1-q_{1}\right)q_{2}p_{2/2}+\mathrm{Pr}\left\{Q\neq 0\right\}q_{1}q_{2}p_{2/1,2}\\ \; & =q_{2}\left(1-q_{1}\mathrm{Pr}\left\{Q\neq 0\right\}\right)p_{2/2}+q_{2}q_{1}\mathrm{Pr}\left\{Q\neq 0\right\}p_{2/1,2}. \end{array}$$

The status updates at the first user are arriving according to a Bernoulli process with a probability λ. When the status update queue of the first user is stable $\lambda <\mu_{1}$, the probability that the queue of user 1 is not empty can be written as

(7) $$\begin{array}{c} \mathrm{Pr}\left\{Q\neq 0\right\}=\frac{\lambda }{\mu_{1}}. \end{array}$$

Now, using (7), the expression (6) can be written as

(8) $$\begin{array}{c} \mu_{2}=q_{2}\left(p_{2/2}-\frac{\lambda (p_{2/2}-p_{2/1,2})}{p_{1/1}-q_{2}\left(p_{1/1}-p_{1/1,2}\right)}\right). \end{array}$$

## 3. Analysis of the Age of Information and Problem Formulation

In this section, we analyze the average AoI of the first and second users, and formulate an optimization problem to minimize the average AoI of the first user. In the following subsections, we first derive the average AoI of the first user for a discrete-time FCFS queue, LCFS queue with preemption, and queue with packet replacement policies. Then, we obtain the AoI and average AoI of the second user for a case threshold policy.

### 3.1. Average Age of Information of the First User

**Figure 2:**
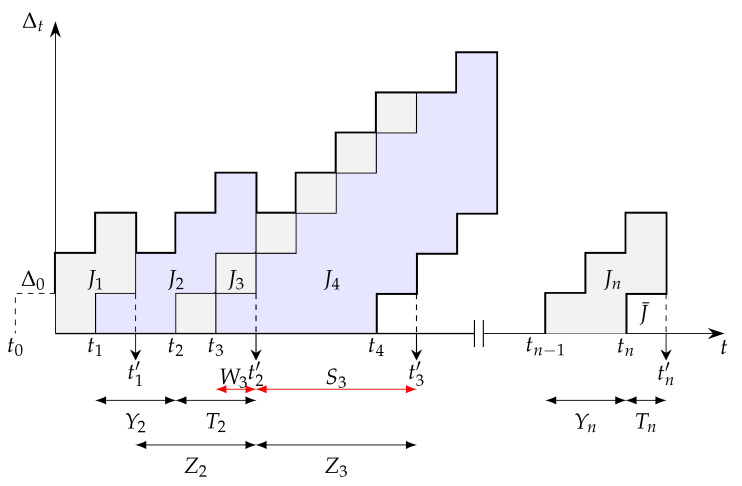
An example of the age evolution of user 1 at the receiver.

The AoI of the first user at the receiver is defined as a random process $\Delta_{t}=t-G\left(t\right)$, where G(t) is the time slot when the latest successfully received a status update from the first user. The evolution of AoI of the first user is illustrated in Figure 2. In this figure, we assume that all packets need to be delivered to the destination regardless of the freshness of the status update information. Therefore, we consider that *j*th status update is generated at time slot $t_{j}$, and received by the receiver at time slot $t_{j}^{{}^{\prime }}$. Then, we denote $T_{j}=t_{j}^{{}^{\prime }}-t_{j}$ and $Y_{j}=t_{j}-t_{j-1}$ as the system time of update *j* and the interarrival time of update *j*, respectively. Without loss of generality, the average AoI of the first user for an interval of observation (0,τ) is defined as

(9) $$\begin{array}{c} \Delta_{\tau }=\frac{1}{\tau }\sum_{t=0}^{N(\tau )}\Delta_{t}, \end{array}$$

where N(τ) is the number of samples during the observation interval. Using Figure 2, Equation (9) can be calculated as the area under $\Delta_{t}$. Starting from t=0, the area is decomposed into the areas $J_{1},J_{2},\ldots ,J_{N(\tau )}$, and the area of width $T_{n}$ over the time interval $\left(t_{n},t_{n}^{\prime }\right)$ that is denoted by $\bar{J}$. Therefore, one can write the average AoI of the first user as a sum of disjoint geometric parts as

(10) $$\begin{array}{c} \Delta_{\tau }=\frac{1}{\tau }\left(J_{1}+\bar{J}+\sum_{j=2}^{N(\tau )}J_{j}\right)=\frac{J_{1}+\bar{J}}{\tau }+\frac{N(\tau )-1}{\tau }\frac{1}{N(\tau )-1}\sum_{j=2}^{N(\tau )}J_{j}. \end{array}$$

Now, the average AoI of the first user is given by

(11) $$\begin{array}{c} {\bar{A}}_{1}=\lim_{\tau \to \infty }\Delta_{\tau }, \end{array}$$

we can write the expression given in (11) as [5]

(12) $$\begin{array}{c} {\bar{A}}_{1}=\frac{1}{E[Y]}\left(E\left[YT\right]+\frac{E[Y^{2}]}{2}+\frac{E[Y]}{2}\right). \end{array}$$

### 3.2. The FCFS Geo/Geo/1 Queue

In this section, we obtain the average AoI of the first user for a discrete-time Geo/Geo/1 queue discipline of FCFS. When status update packets are arriving according to the Bernoulli process with a probability λ, the interarrival times $Y_{j}$ are i.i.d. sequences that follow a geometric distribution with probability mass function (PMF) as

(13) $$\begin{array}{c} \mathrm{Pr}\left\{Y_{j}=y\right\}=\lambda {\left(1-\lambda \right)}^{y-1},y=1,2,\ldots . \end{array}$$

Thus, we can obtain E[Y] and $E[Y^{2}]$ in Equation (12) as

(14) $$\begin{array}{c} E\left[Y\right]=\frac{1}{\lambda },\;E\left[Y^{2}\right]=\frac{2-\lambda }{\lambda^{2}}. \end{array}$$

Also, the expression E[YT] can be obtained as [13]

(15) $$\begin{array}{c} E\left[YT\right]=\frac{\lambda (1-\mu_{1})}{\left(\mu_{1}-\lambda \right)\mu_{1}^{2}}+\frac{1}{\lambda \mu_{1}}. \end{array}$$

Now, using Equations (14) and (15), the expression given in (12) can be written as

(16) $$\begin{array}{c} {\bar{A}}_{1}=\frac{1}{\lambda }+\frac{1-\lambda }{\mu_{1}-\lambda }-\frac{\lambda }{\mu_{1}^{2}}+\frac{\lambda }{\mu_{1}}. \end{array}$$

### 3.3. The Preemptive LCFS Geo/Geo/1 Queue

In this section, we consider a discrete-time LCFS Geo/Geo/1 queue with preemptive service, where a newly generated packet is given priority for service immediately. It is assumed that status update packets are arriving according to the Bernoulli process with probability λ. The probability distribution of interarrival time between the *j*th and (j+1)th status update packet is assumed to be geometric with mean E[Y]=1/λ, and the probability distribution time until successful delivery is assumed to be geometric distribution with mean $E\left[S\right]=1/\mu_{1}$, in which $\mu_{1}$ denotes the service probability of the first user. In [32], it is shown that the PMF of AoI for a discrete-time LCFS Geo/Geo/1 queue is given by

(17) $$\begin{array}{c} \mathrm{Pr}\left\{A_{1}=x\right\}=\frac{\lambda \mu_{1}\left[{\left(1-\lambda \right)}^{x-1}-{\left(1-\mu_{1}\right)}^{x-1}\right]}{\mu_{1}-\lambda }. \end{array}$$

Now, we can write the average AoI of the first user as

(18) $$\begin{array}{c} {\bar{A}}_{1}=\sum_{x=1}^{\infty }x\mathrm{Pr}\left\{A_{1}=x\right\}=\frac{1}{\lambda }+\frac{1}{\mu_{1}}. \end{array}$$

### 3.4. Queue with Replacement

In this section, we derive the average AoI of the first user for a queue with replacement. In this case, it is assumed that a newly generated packet discards the packet waiting in the queue. As shown in Figure 2, we express the areas $J_{j}$ with respect to the random variables $Z_{j}$ as follows

(19) $$\begin{array}{cc} J_{j} & =\sum_{m=1}^{T_{j-1}+Z_{j}}m-\sum_{m=1}^{T_{j}}m\\ \; & =\frac{\left(T_{j-1}+Z_{j}\right)\left(T_{j-1}+Z_{j}+1\right)}{2}-\frac{T_{j}\left(T_{j}+1\right)}{2}. \end{array}$$

We use the fact that in the steady state $T_{j-1}$ and $T_{j}$ are identically distributed. Therefore, the average AoI of the first user for a queue with packet replacement is given by [13]

(20) $$\begin{array}{c} {\bar{A}}_{1}=\lambda_{e}\left(E\left[ZT\right]+\frac{E[Z^{2}]}{2}+\frac{E[Z]}{2}\right), \end{array}$$

where one can obtain $\lambda_{e}$, E[Z], $E[Z^{2}]$ and E[ZT] as [13]

(21) $$\begin{array}{cc} \lambda_{e} & =\lambda -\frac{\lambda^{3}\left(1-\mu_{1}\right)}{\lambda^{2}\left(1-\mu_{1}\right)+\lambda \left(1-\mu_{1}\right)\mu_{1}+\mu_{1}^{2}}\\ E[Z] & =\frac{\lambda^{2}\left(1-\mu_{1}\right)+\lambda \left(1-\mu_{1}\right)\mu_{1}+\mu_{1}^{2}}{\lambda \mu_{1}\left(\lambda +\mu_{1}-\lambda \mu_{1}\right)}\\ E[Z^{2}] & =\frac{\left(2\lambda^{2}+2\lambda \mu_{1}-\lambda^{2}\mu_{1}+2\mu_{1}^{2}-\lambda \mu_{1}^{2}\right)\left(\mu_{1}-\lambda \mu_{1}\right)}{\lambda^{2}\mu_{1}^{2}\left(\lambda +\mu_{1}-\lambda \mu_{1}\right)}+\frac{\lambda (2-\mu_{1})}{\mu_{1}^{2}\left(\lambda +\mu_{1}-\lambda \mu_{1}\right)}\\ E[ZT] & =\frac{1}{\mu_{1}^{2}}+\frac{1-\lambda }{\lambda \mu_{1}}-\frac{1+\lambda }{{\left(\lambda +\mu_{1}-\lambda \mu_{1}\right)}^{2}}+\frac{1+2\lambda }{\lambda +\mu_{1}-\lambda \mu_{1}}+\frac{\lambda (1-2\mu_{1}+\lambda \left(3\mu_{1}-2\right))}{\lambda^{2}{\left(1-\mu_{1}\right)}^{2}+\lambda \mu_{1}\left(1-2\mu_{1}\right)+\mu_{1}^{2}}. \end{array}$$

### 3.5. Age of Information and the Average Age of Information of the Second User

We assume $A_{2}\left(t\right)$ be a positive integer that represents the AoI associated with the second user at the receiver. The AoI evolution between two consecutive time slots at the receiver can be written as

(22) $$A_{2}\left(t+1\right)=\left\{\begin{array}{c} 1,\;\mathrm{successful}\;\mathrm{packet}\;\mathrm{reception}\;\mathrm{at}\;\mathrm{time}\;\mathrm{slot}\;t\\ A_{2}\left(t\right)+1,\;\mathrm{otherwise}. \end{array}\right.$$

**Figure 3:**
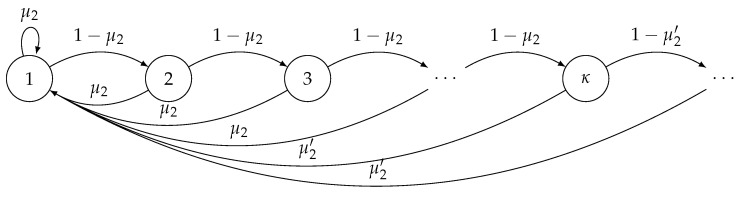
The DTMC, which models the evolution of AoI of the second user.

According to (22), the AoI drops to one when there is a successful reception of a status update at the receiver. Otherwise, it increases by one. We can model the evolution of the AoI of the second user as a Discrete-Time Markov Chain (DTMC). The DTMC is shown in Figure 3, in which when $A_{2}\left(t\right)<\kappa$ (κ is the threshold of the AoI of the second user), a packet is transmitted with the probability $q_{2}$. Also, a packet is transmitted with the probability $q_{2}^{\prime }$ when $A_{2}\left(t\right)⩾\kappa$. The service probability of the first user can be written as

(23) $$\begin{array}{c} \mu_{1}^{\prime }=q_{1}\left(1-q_{2}^{\prime }\right)p_{1/1}+q_{1}q_{2}^{\prime }p_{1/1,2}. \end{array}$$

According to the DTMC described in Figure 3, we can obtain the steady-state probabilities of the AoI of the second user as follows

(24) $$\begin{array}{cc} \pi_{i} & =\left\{\;\;\begin{array}{cc} {\left(1-\mu_{2}\right)}^{i-1}\pi_{1} & ,i<\kappa \\ {\left(\frac{1-\mu_{2}}{1-\mu_{2}^{\prime }}\right)}^{\kappa -1}{\left(1-\mu_{2}^{\prime }\right)}^{i-1}\pi_{1} & ,i⩾\kappa \end{array}\right. \end{array}$$

where for $\lambda <\mu_{1}^{\prime }$, $\mu_{2}^{\prime }$ is given by

(25) $$\begin{array}{c} \mu_{2}^{\prime }=q_{2}^{\prime }\left(p_{2/2}-\frac{\lambda (p_{2/2}-p_{2/1,2})}{p_{1/1}-q_{2}^{\prime }\left(p_{1/1}-p_{1/1,2}\right)}\right). \end{array}$$

Additionally, we can obtain $\pi_{1}$ as

(26) $$\begin{array}{cc} \pi_{1} & =\left\{\;\;\begin{array}{cc} \mu_{2}^{\prime } & ,\kappa =1\\ \frac{\mu_{2}\mu_{2}^{\prime }}{\mu_{2}^{\prime }+\left(\mu_{2}-\mu_{2}^{\prime }\right){\left(1-\mu_{2}\right)}^{\kappa -1}} & ,\kappa ⩾2. \end{array}\right. \end{array}$$

Using Equations (24) and (26), we can write the probability that the AoI of the second user is smaller than a threshold κ, as follows

(27) $$\begin{array}{c} \mathrm{Pr}\left\{A_{2}<\kappa \right\}=\sum_{i=1}^{\kappa -1}{\left(1-\mu_{2}\right)}^{i-1}\pi_{1}=\frac{\left(1-{\left(1-\mu_{2}\right)}^{\kappa -1}\right)\pi_{1}}{\mu_{2}}. \end{array}$$

Furthermore, one can write the probability that the AoI of the second user is greater than a threshold, κ, as follows

(28) $$\begin{array}{cc} \mathrm{Pr}\{A_{2}⩾\kappa \} & =\sum_{i=\kappa }^{\infty }{\left(\frac{1-\mu_{2}}{1-\mu_{2}^{\prime }}\right)}^{\kappa -1}{\left(1-\mu_{2}^{\prime }\right)}^{i-1}\pi_{1}\\ \; & =\frac{{\left(1-\mu_{2}\right)}^{\kappa -1}\pi_{1}}{\mu_{2}^{\prime }}. \end{array}$$

Now, using Equations (27) and (28), the average AoI of the second user is described as

(29) $$\begin{array}{cc} {\bar{A}}_{2} & =\sum_{i=1}^{\infty }i\pi_{i}=\sum_{i=1}^{\kappa -1}i{\left(1-\mu_{2}\right)}^{i-1}\pi_{1}+\sum_{i=\kappa }^{\infty }i{\left(\frac{1-\mu_{2}}{1-\mu_{2}^{\prime }}\right)}^{\kappa -1}{\left(1-\mu_{2}^{\prime }\right)}^{i-1}\pi_{1}\\ \; & =\frac{1-\mu_{2}-{\left(1-\mu_{2}\right)}^{\kappa }\left[1-\mu_{2}+\kappa \mu_{2}\right]}{\left(1-\mu_{2}\right)\mu_{2}^{2}}\pi_{1}+\frac{{\left(1-\mu_{2}\right)}^{\kappa }\left[1-\mu_{2}^{\prime }+y\mu_{2}^{\prime }\right]}{\left(1-\mu_{2}\right)\mu_{2}^{\prime 2}}\pi_{1}, \end{array}$$

where $\mu_{2}$, $\mu_{2}^{\prime }$, and $\pi_{1}$ are given by (8), (25) and (26), respectively. Additionally, when κ→∞ and $\lambda <\mu_{1}$, Equation (29) can be written as

(30) $$\begin{array}{c} {\bar{A}}_{2}=\frac{1}{\mu_{2}}. \end{array}$$

### 3.6. The Average AoI of $S_{1}$ for the Preemptive LCFS Geo/Geo/1 Queue for the Threshold-Based Policy of $S_{2}$

By considering the threshold-based policy explained in Section 3.5, the average AoI of $S_{1}$ for the preemptive LCFS queue discipline given in (17) can be written as

(31) $$\begin{array}{cc} \mathrm{Pr}\{A_{1}=x\} & =\mathrm{Pr}\left\{A_{1}=x|A_{2}<\kappa \right\}\mathrm{Pr}\left\{A_{2}<\kappa \right\}+\mathrm{Pr}\left\{A_{1}=x|A_{2}⩾\kappa \right\}\mathrm{Pr}\left\{A_{2}⩾\kappa \right\}, \end{array}$$

where $\mathrm{Pr}\{A_{2}<\kappa \}$, $\mathrm{Pr}\{A_{2}⩾\kappa \}$ are given by (27) and (28). Using Equation (17), the first and second conditional probabilities given in (31) can be written as

(32a) $$\begin{array}{c} \mathrm{Pr}\left\{A_{1}=x|A_{2}<\kappa \right\}=\frac{\lambda \mu_{1}\left[{\left(1-\lambda \right)}^{x-1}-{\left(1-\mu_{1}\right)}^{x-1}\right]}{\mu_{1}-\lambda } \end{array}$$

(32b) $$\begin{array}{c} \mathrm{Pr}\left\{A_{1}=x|A_{2}⩾\kappa \right\}=\frac{\lambda \mu_{1}^{\prime }\left[{\left(1-\lambda \right)}^{x-1}-{\left(1-\mu_{1}^{\prime }\right)}^{x-1}\right]}{\mu_{1}^{\prime }-\lambda }, \end{array}$$

where $\mu_{1}^{\prime }$ is given by (23). Now, we can write the average AoI of $S_{1}$ for threshold-based policy of the AoI of $S_{2}$ as

(33) $$\begin{array}{cc} {\bar{A}}_{1} & =\sum_{x=1}^{\infty }x\mathrm{Pr}\left\{A_{1}=x\right\}\\ \; & =\left(\frac{1}{\lambda }+\frac{1}{\mu_{1}}\right)\left(\frac{\left[1-{\left(1-\mu_{2}\right)}^{\kappa -1}\right]\pi_{1}}{\mu_{2}}\right)+\left(\frac{1}{\lambda }+\frac{1}{\mu_{1}^{\prime }}\right)\left(\frac{{\left(1-\mu_{2}\right)}^{\kappa -1}\pi_{1}}{\mu_{2}^{\prime }}\right), \end{array}$$

where $\pi_{1}$ is given by (26).

### 3.7. Optimizing the Average AoI of $S_{1}$ subject to AoI constraints on $S_{2}$

#### 3.7.1. Using the Average AoI of the FCFS as the objective function

In this section, our objective is to minimize the average AoI of user 1 for a discrete-time Geo/Geo/1 queue discipline of FCFS with a constraint on the average AoI for user 2, which should be less than a threshold. Let $A_{\max}$ be a strictly positive real value that represents the maximum average AoI of user 2. Thus, the optimization problem is formulated as follows

(34a) $$\begin{array}{cc} \; & \mathrm{minimize}\;{\bar{A}}_{1} \end{array}$$

(34b) $$\begin{array}{cc} \; & \mathrm{subject}\;\mathrm{to}\;{\bar{A}}_{2}<A_{\max}. \end{array}$$

Using the expressions given in Equations (16) and (30), one can write Equation (34) as follows

(35a) $$\begin{array}{cc} \underset{q_{1},q_{2},\lambda }{\mathrm{minimize}}\; & \frac{1}{\lambda }+\frac{1-\lambda }{\mu_{1}-\lambda }-\frac{\lambda }{\mu_{1}^{2}}+\frac{\lambda }{\mu_{1}} \end{array}$$

(35b) $$\begin{array}{cc} \mathrm{subject}\;\mathrm{to}\; & \frac{1}{\mu_{2}}<A_{\max}, \end{array}$$

(35c) $$\begin{array}{cc} \; & 0⩽\lambda <\mu_{1}, \end{array}$$

(35d) $$\begin{array}{cc} \; & q_{1},q_{2}\in \left[0,1\right]. \end{array}$$

where the constraint in (35c) ensures that the queue of the first user is stable. To solve this optimization problem, we first note that for $\lambda <\mu_{1}$ when the service probability of the first user increases, the objective function given in (35a) decreases. Hence, to minimize the objective function, we must obtain the maximum value of $\mu_{1}$. The service probability of the first user given in (5) can be simplified as

(36) $$\begin{array}{cc} \mu_{1} & =q_{1}\left(1-q_{2}\right)p_{1/1}+q_{1}q_{2}p_{1/1,2}\\ \; & =q_{1}\left[p_{1/1}-q_{2}\left(p_{1/1}-p_{1/1,2}\right)\right]. \end{array}$$

According to Equation (36), $\mu_{1}$ has its maximum value when $q_{1}$ is maximum. Therefore, by selecting $q_{1}=1$, we can maximize the service probability of the first user and minimize the average AoI of the first user as an objective function. Therefore, the optimal value of $q_{1}$ is given by

(37) $$\begin{array}{cc} q_{1}^{*} & =1. \end{array}$$

Now, using Equations (8), (36) and (37), we can write the optimization problem given in (35) as

(38a) $$\begin{array}{cc} \underset{q_{2},\lambda }{\mathrm{minimize}}\; & \frac{1}{\lambda }+\frac{1-\lambda }{p_{1/1}-q_{2}\left(p_{1/1}-p_{1/1,2}\right)-\lambda }-\frac{\lambda \left(1-p_{1/1}+q_{2}\left(p_{1/1}-p_{1/1,2}\right)\right)}{{\left(p_{1/1}-q_{2}\left(p_{1/1}-p_{1/1,2}\right)\right)}^{2}} \end{array}$$

(38b) $$\begin{array}{cc} \mathrm{subject}\;\mathrm{to}\; & \frac{p_{1/1}-q_{2}\left(p_{1/1}-p_{1/1,2}\right)}{q_{2}\left(p_{1/1}p_{2/2}-q_{2}p_{2/2}\left(p_{1/1}-p_{1/1,2}\right)-\lambda \left(p_{2/2}-p_{2/1,2}\right)\right)}-A_{\max}<0, \end{array}$$

(38c) $$\begin{array}{cc} \; & \lambda -p_{1/1}+q_{2}\left(p_{1/1}-p_{1/1,2}\right)<0, \end{array}$$

(38d) $$\begin{array}{cc} \; & \lambda ,q_{2}\in \left[0,1\right]. \end{array}$$

By definition, an optimization problem is convex when its objective function and the inequality constraints are convex, and its equality constraints are affine, see Chapter 4.2 in [33]. We can show that the Hessian matrix of the objective function given in (38a) is positive semi-definite for some parameters of λ and $q_{2}$ and for some others is not positive semi-definite and therefore it is not a convex function. Additionally, it can be verified that the Hessian matrices of the inequality constraints (38b) and (38c) are positive semi-definite for different values of λ and $q_{2}$. Therefore, this optimization problem is not a convex optimization problem, a trivial solution does not exist for this problem, and finding the optimal solution is computationally involved. Hence, to find the optimal values of λ and $q_{2}$, a suboptimal technique is proposed to effectively solve the problem using an algorithm developed for solving convex optimization problems. This approach is known as the bilevel optimization algorithm and is used when optimization parameters are interdependent, and the optimization problem is convex with respect to each of the optimization parameters when other parameters are fixed [34].

#### 3.7.2. Bilevel Convex Optimization

Using the procedure explained in Appendix A, it can be verified that the objective function given in (38a) is a convex function of λ when $q_{2}$ is fixed and $\lambda <\mu_{1}$. Therefore, the optimization problem can be solved for λ by assuming that $q_{2}$ is fixed. Then, substituting for λ in (38a) from the previous stage and assuming that this parameter is fixed, we can solve the optimization problem for $q_{2}$. This procedure continues until the convergence condition is satisfied (for example, the change in the objective function in two successive iterations is lower than a small threshold).

In this paper, an interior-point method is used to solve the optimization problem in each iteration of the bilevel optimization algorithm. The iteration complexity of this method is shown in Chapter 3.4.3 in the work of den Hertog [35] to be $O(\nu \left(c\sqrt{n}\right))$, where ν denotes the number of iterations, *n* is the number of constraints and *c* is a constant, which depends on system parameters such as tolerance.

#### 3.7.3. Using the Average AoI of the LCFS with Preemption as an Objective Function

In this section, our objective is to minimize the average AoI of user 1 for a discrete-time preemptive LCFS Geo/Geo/1 queue discipline with a constraint on the average AoI for user 2, which should be less than a threshold. Using the expressions given in Equations (18) and (30), the optimization problem is formulated as follows

(39a) $$\begin{array}{cc} \underset{q_{1},q_{2},\lambda }{\mathrm{minimize}}\; & \frac{1}{\lambda }+\frac{1}{\mu_{1}} \end{array}$$

(39b) $$\begin{array}{cc} \mathrm{subject}\;\mathrm{to}\; & \frac{1}{\mu_{2}}<A_{\max}, \end{array}$$

(39c) $$\begin{array}{cc} \; & 0⩽\lambda <\mu_{1}, \end{array}$$

(39d) $$\begin{array}{cc} \; & q_{1},q_{2}\in \left[0,1\right]. \end{array}$$

Using the procedure explained in Section 3.7.1, the $q_{1}^{*}=1$ and the optimization problem given in (39) is simplified as

(40a) $$\begin{array}{cc} \underset{q_{2},\lambda }{\mathrm{minimize}}\; & \frac{1}{\lambda }+\frac{1}{p_{1/1}-q_{2}\left(p_{1/1}-p_{1/1,2}\right)} \end{array}$$

(40b) $$\begin{array}{cc} \mathrm{subject}\;\mathrm{to}\; & \frac{p_{1/1}-q_{2}\left(p_{1/1}-p_{1/1,2}\right)}{q_{2}\left(p_{1/1}p_{2/2}-q_{2}p_{2/2}\left(p_{1/1}-p_{1/1,2}\right)-\lambda \left(p_{2/2}-p_{2/1,2}\right)\right)}-A_{\max}<0, \end{array}$$

(40c) $$\begin{array}{cc} \; & \lambda -p_{1/1}+q_{2}\left(p_{1/1}-p_{1/1,2}\right)<0, \end{array}$$

(40d) $$\begin{array}{cc} \; & \lambda ,q_{2}\in \left[0,1\right]. \end{array}$$

We can prove that the Hessian matrix of the objective function given in (40a) is positive semi-definite and the optimization problem is convex (see Appendix B). Therefore, this optimization problem can be solved using an algorithm developed for solving convex optimization problems such as the interior-point method.

## 4. Numerical Results and Discussion

In this section, we illustrate our analytical results presented in Section 3 and we verify them by means of computer simulation. Simulation results are obtained using $10^{6}$ independent realizations of the system. Additionally, we evaluate the performance of the proposed interior-point algorithm presented in Section 3. It is assumed that the users are located at a distance $r_{i}=30\;m$ (*i* = 1, 2) from the receiver. The receiver noise power is assumed to be $\sigma^{2}=-100\;\mathrm{dBm}$, and the path-loss exponent is α=4. Additionally, the assumed transmit powers are $P_{t,1}=P_{t,2}=5\;\mathrm{mW}$, and the transmission channels between the users and receiver are subject to Rayleigh fading model and we use the expressions for the success probabilities that were presented in Section 2. Furthermore, the initial point for the interior-point algorithm is zero, i.e., $\left(\lambda^{\left(0\right)},q_{2}^{\left(0\right)}\right)=0$.

**Figure 4:**
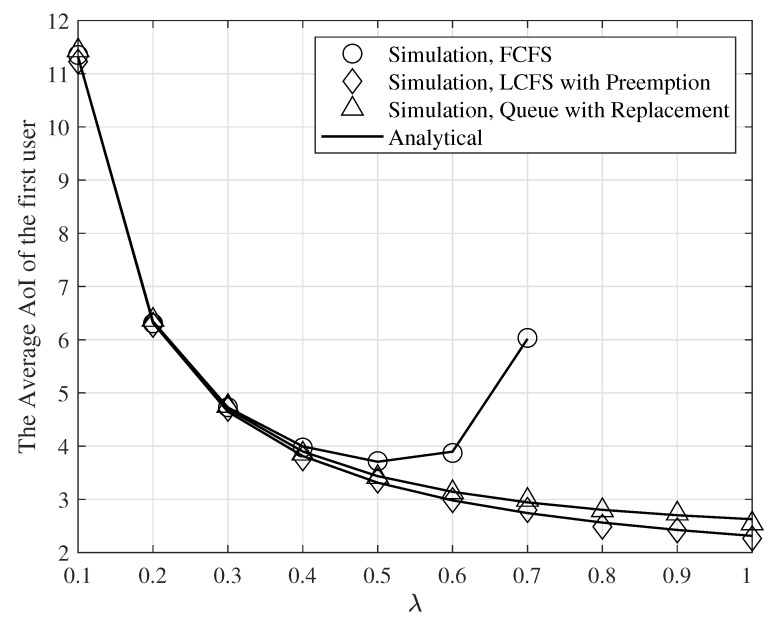
The average AoI of the first user for the FCFS Geo/Geo/1 queue, preemptive LCFS Geo/Geo/1 queue, and queue with replacement for γ=−5dB, M=1, $q_{1}=0.8$, and $q_{2}=0.2$, and various values of λ.

Figure 4 shows the average AoI of the first user for the FCFS Geo/Geo/1 queue, preemptive LCFS Geo/Geo/1 queue, and queue with replacement as a function of λ, γ=−5dB, M=1, $q_{1}=0.8$, and $q_{2}=0.2$. As seen in this figure, the preemptive LCFS Geo/Geo/1 queue outperforms the FCFS Geo/Geo/1 queue, and queue with replacement. Additionally, note that the average AoI of the first user for the FCFS Geo/Geo/1 queue we plot for λ<0.7 to satisfy the stability requirements. Figure 4 also shows that the simulation results match the analytical results.

**Figure 5:**
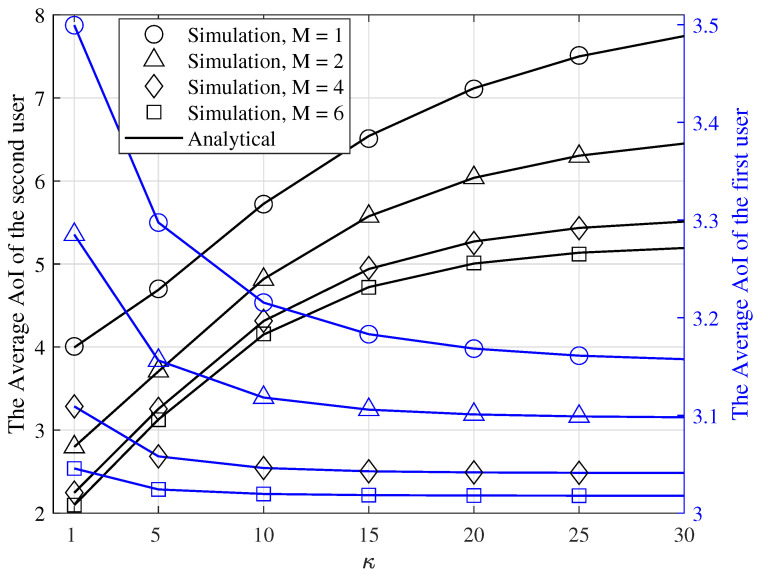
The average AoI of the $S_{1}$ and $S_{2}$ for γ=3dB, λ=0.5, $q_{1}=1$, $q_{2}=0.2$, $q_{2}^{\prime }=0.5$, κ=1,5,10,⋯,30, and various values of *M*.

The average AoI of $S_{1}$ and $S_{2}$ are shown in Figure 5 as a function of κ, for λ=0.5, $q_{1}=1$, $q_{2}=0.2$, $q_{2}^{\prime }=0.5$, γ=3dB, and various values of *M*. As seen in this figure, as κ increases, the slope of the average AoI of $S_{2}$ and $S_{1}$ decreases because when κ becomes larger, the average AoI of $S_{2}$ and $S_{1}$ tends to $1/\mu_{2}$ and $1/\lambda +1/\mu_{1}$, respectively, and becomes independent of $q_{2}^{\prime }$. Therefore, by changing $q_{2}^{\prime }$ the average AoI of $S_{2}$ and $S_{1}$ will not change. Furthermore, when κ increases, the average AoI of $S_{2}$ increases and the average AoI of $S_{1}$ decreases. This is because for smaller values of κ, a packet is transmitted with probability $q_{2}^{\prime }$ that $q_{2}^{\prime }>q_{2}$ sooner than larger values of κ. Therefore, the average AoI of the first and second users has larger and smaller values, respectively, for smaller values of κ. Additionally, note that when *M* increases, the average AoI of the first and second users decreases because for a larger value of *M* the service probabilities of the first and second users have larger values such that they decrease the average AoI of $S_{1}$ and $S_{2}$.

**Figure 6:**
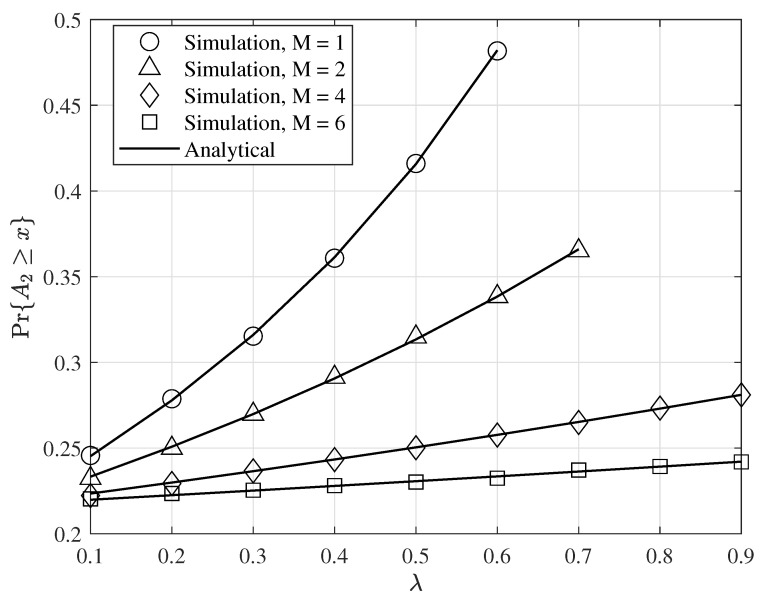
The probability the AoI of the $S_{2}$ at the receiver greater than a threshold, x=5, for γ=3dB, $q_{1}=1$, $q_{2}=0.2$, $q_{2}^{\prime }=0.5$, λ=0.1,0.2,⋯,1, and various values of *M*.

Figure 6 shows the probability that the AoI of the second user to be greater than a threshold as a function of λ for $q_{1}=1$, $q_{2}=0.2$, $q_{2}^{\prime }=0.5$, γ=3dB, x=5, and selected values of *M*. As seen in this figure, when λ increases the probability $\mathrm{Pr}\{A_{2}⩾x\}$ increases. This is because when λ increases the service probability of the second user decreases and therefore it increases the AoI of the second user. Furthermore, by increasing *M*, the success transmission probabilities increase, and the service probability of the second user increases. As a result, the AoI of the second user decreases and the probability $\mathrm{Pr}\{A_{2}⩾x\}$ has lower values. Note, importantly, that when M=1, the probability $\mathrm{Pr}\{A_{2}⩾x\}$ does not have a value for λ>0.6. This is because for λ>0.6, λ becomes larger than $\mu_{1}$ and thus the AoI of the second user does not have a value.

**Figure 7:**
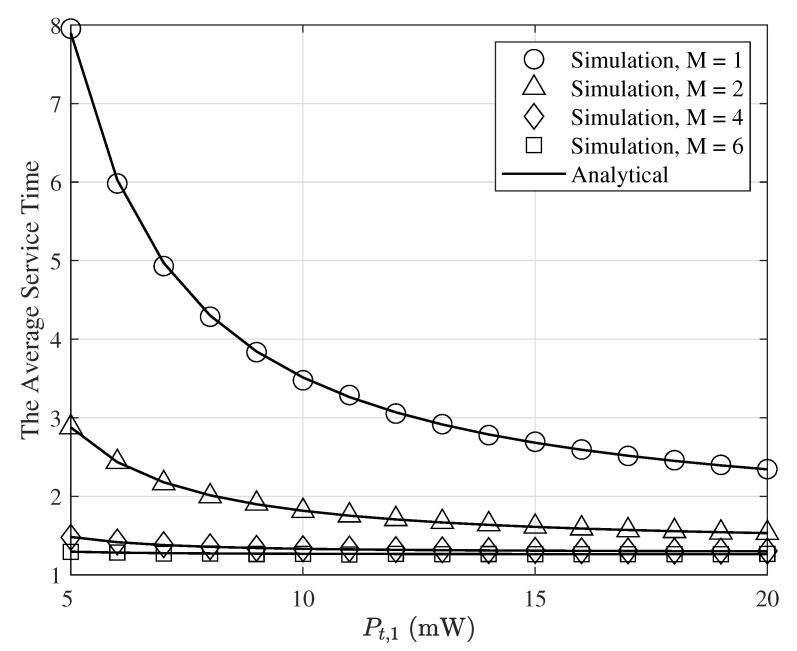
The average service time of the first user as a function of $P_{t,1}$ for $\sigma^{2}=-50\;\mathrm{dBm}$, α=4, $r_{1}=30\;m\;\left(i=1,2\right)$, γ=0dB, $q_{1}=0.8$, $q_{2}=0.4$, and various values of *M*.

Figure 7 shows the average service time of the first user, $1/\mu_{1}$, as a function of $P_{t,1}\;$($P_{t,1}=P_{t,2}$) for $\sigma^{2}=-50\;\mathrm{dBm}$, α=4, $r_{i}=30\;m\;$ (*i* = 1, 2), γ=0dB, $q_{1}=0.8$, $q_{2}=0.4$, and selected values of *M*. As seen in this figure, when transmitted power increases, the average service time decreases. This is because by increasing the transmitted power, the success transmission probabilities increases and thus the average service time decreases. Furthermore, when *M* increases, the average service time decreases. This is because when *M* increases, the service probability increases such that it decreases the average service time.

**Figure 8:**
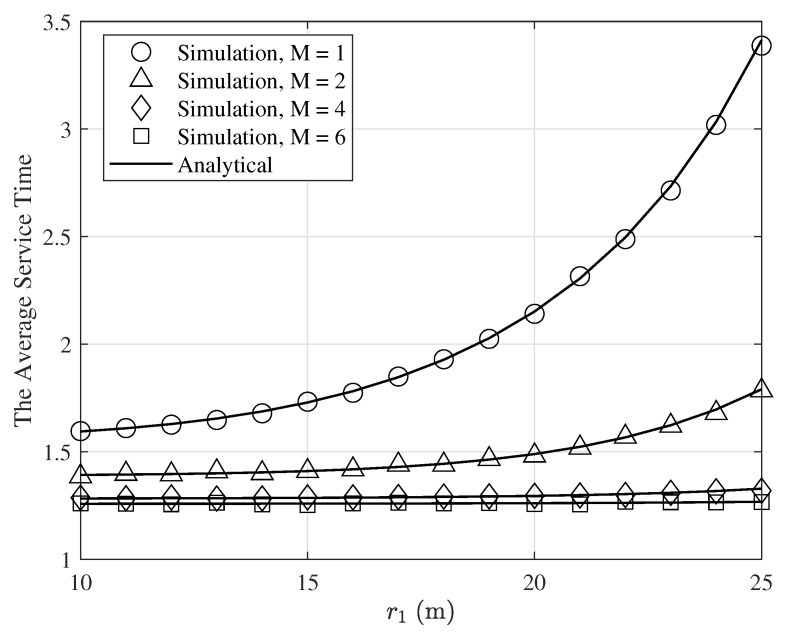
The average service time of the first user as a function of $r_{1}$ for $\sigma^{2}=-50\;\mathrm{dBm}$, α=4, $P_{t,1}=P_{t,2}=5\;\mathrm{mW}$, γ=0dB, $q_{1}=0.8$, $q_{2}=0.4$, and various values of *M*.

The average service time is illustrated in Figure 8 as a function of $r_{1}\;$($r_{1}=r_{2}$) for $\sigma^{2}=-50\;\mathrm{dBm}$, α=4, $P_{t,1}=P_{t,2}=5\;\mathrm{mW}$, γ=0dB, $q_{1}=0.8$, $q_{2}=0.4$, and various values of *M*. As seen in this figure, the average service time increases by increasing $r_{1}$. This is because when $r_{1}$ increases, the service probability of the first user decreases, which results in decreasing in the average service time. Moreover, by increasing *M*, the average service time decreases. This is because when *M* increases, the success probabilities increase and therefore the average service time decreases.

**Figure 9:**
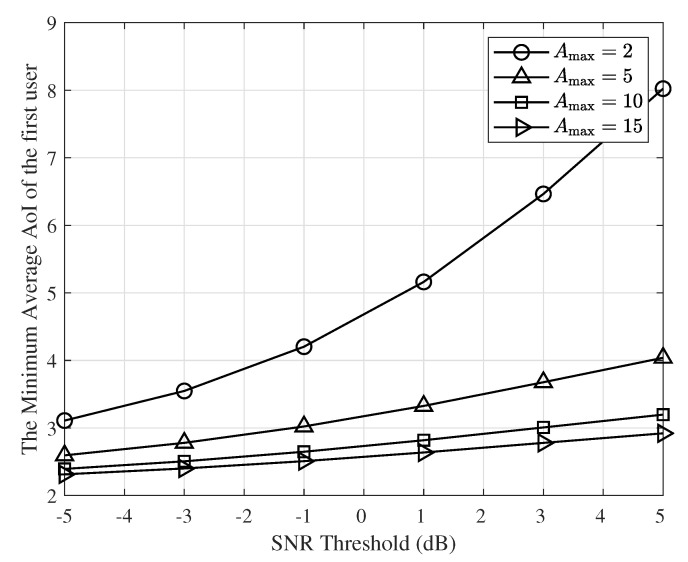
The minimum average AoI of user 1, for M=1, $A_{\max}=2,5,10,15$, and various values of γ.

The minimum average AoI of the first user for the case where M=1 as a function of γ and selected values of $A_{\max}$ is illustrated in Figure 9. As seen in this figure and Table 1, Table 2, Table 3 and Table 4, the minimum average AoI of the first user has a larger value when the SNR threshold γ is larger. This is because a higher γ gives lower success probabilities and therefore increases the minimum average AoI. An important observation is that as $A_{\max}$ increases, the average AoI of the first user does not depend on $A_{\max}$. This is because when $A_{\max}$ increases, the constraint on the average AoI of the second user becomes independent of $A_{\max}$. Hence, when $A_{\max}$ increases, the optimal value of transmit probability $q_{2}$, λ and therefore the minimum average AoI of the first user will not change.

**Figure 10:**
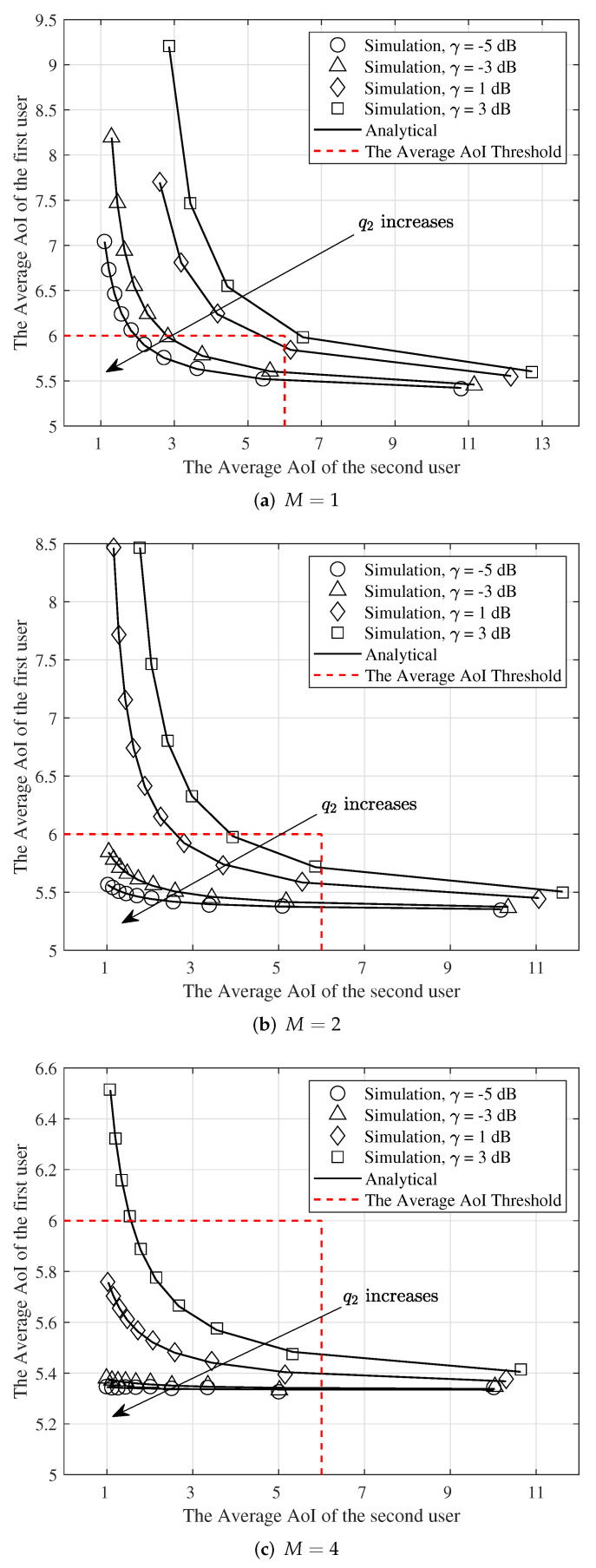
The interplay between the average AoI of the first and second users for $q_{1}=0.6$, λ=0.3, $q_{2}=0.1,0.2,\ldots ,1$, (**a**) M=1, (**b**) M=2, and (**c**) M=4.

Figure 10 shows the interplay between the average AoI of the first user for a discrete-time Geo/Geo/1 queue discipline of FCFS and the average AoI of the second user when y→∞ as a function of $q_{2}$ and selected values of γ. In this figure, we consider the weak/strong MPR capabilities. We denote that the strong and weak MPR capability of a receiver corresponds to $K=\frac{p_{1/1,2}}{p_{1/1}}+\frac{p_{2/1,2}}{p_{2/2}}>1$ and $K=\frac{p_{1/1,2}}{p_{1/1}}+\frac{p_{2/1,2}}{p_{2/2}}<1$, respectively [24]. When M=1 for γ=−5dB and γ=−3dB, K=1.51 and K=1.33, respectively. Therefore, the receiver has strong MPR capabilities. Furthermore, for γ=1dB and γ=3dB, K=0.88 and K=0.66, respectively; thus, the receiver has weak MPR capabilities. Additionally, when M=2 for γ∈{−5,−3,1,5}dB, K∈{1.88,1.77,1.37,1.11}, respectively. Moreover, when M=4 for γ∈{−5,−3,1,5}dB, K∈{1.99,1.97,1.80,1.60}, respectively. Therefore, when M>1 the receiver has strong MPR capabilities for selected values of γ. In Figure 10, we consider a different scenario from the optimization problem scenario in (34). Here we intend to find transmission probabilities $q_{1}$ and $q_{2}$ that both users transmit at the same time to keep the average AoI of the first and second users below a threshold $A_{1\max1}$ and $A_{2\max}$ respectively. Observe that when the receiver has strong MPR capabilities, we have ${\bar{A}}_{1}<A_{1\max}$ and ${\bar{A}}_{2}<A_{2\max}$ with a high value of transmit probability $q_{2}$. Therefore, in this case, both users can transmit at the same time with a high probability. For example, we assume the thresholds for the average AoI of the first and second users are equal to $A_{1\max}=6$ and $A_{2\max}=6$, respectively. As seen in this figure when *M* equals 1, and γ=−5dB (strong MPR capability), we can achieve our purpose with $q_{1}=0.6$ and $q_{2}=0.5$ while for γ=1dB (weak MPR capability), we cannot find a value of $q_{2}$ to achieve our goal. In this case, both users cannot transmit at the same time. Additionally, observe that in Figure 10b, when γ=−5dB the first and second users can transmit at the same time with the probabilities of $q_{1}=0.6$ and $q_{2}=1$. Furthermore, when γ=1dB, the transmit probability of $q_{1}$ and $q_{2}$ are equal to $q_{1}=0.6$ and $q_{2}=0.4$. In addition, as shown in Figure 10c, when γ=−5dB and γ=1dB, both users can transmit at the same time with the probabilities of $q_{1}=0.6$ and $q_{2}=1$. This reflects the fact that when the number of receiver antenna increases, the receiver has a strong MPR capability for higher values of γ and thus both users can transmit at the same time with a high probability.

## 5. Discussion on Larger Topology

**Figure 11:**
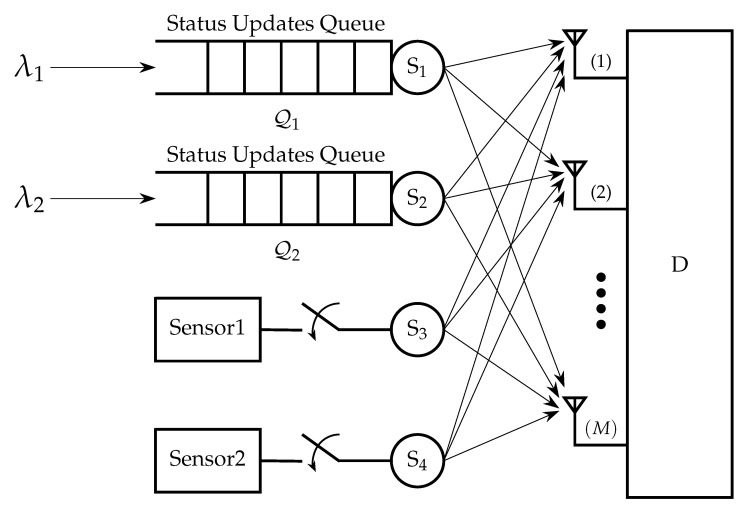
$S_{1}$ and $S_{2}$ have AoI-oriented external bursty traffic, $S_{3}$ and $S_{4}$ have also AoI-oriented traffic but they can control the generation of status updates.

In this section, we discuss how this work can be extended to capture more than two users. However, detailed analysis and optimization for more than two users is left for a future publication. Below, we provide some details for a setup with two users with external traffic and two users with control over the generation of the status updates as depicted in Figure 11. More specifically, it is assumed that the users $S_{1}$ and $S_{2}$ do not have control over the generation of status update packets, and they are externally generated according to Bernoulli processes with probabilities $\lambda_{1}$ and $\lambda_{2}$, respectively. When the queue of $S_{i}$, i=1,2 is not empty, $S_{i}$ attempts to transmit with probability $q_{i}$. We also consider that users $S_{3}$ and $S_{4}$ can control the generation of status update packets; thus, they sample and transmit with probabilities $q_{3}$ and $q_{4}$, respectively, based on a generate-at-will policy. Using the same approach presented in Section 2.2, we derive the service probabilities of $S_{1}$, and $S_{3}$ as

(41) $$\begin{array}{cc} \mu_{1} & =q_{1}\mathrm{Pr}\left\{Q_{2}=0\right\}\left(1-q_{3}\right)\left(1-q_{4}\right)p_{1/1}+q_{1}\mathrm{Pr}\left\{Q_{2}=0\right\}q_{3}\left(1-q_{4}\right)p_{1/1,3}\\ \; & +q_{1}\mathrm{Pr}\left\{Q_{2}=0\right\}\left(1-q_{3}\right)q_{4}p_{1/1,4}+q_{1}\mathrm{Pr}\left\{Q_{2}=0\right\}q_{3}q_{4}p_{1/1,3,4}\\ \; & +q_{1}\mathrm{Pr}\left\{Q_{2}\neq 0\right\}\left(1-q_{2}\right)\left(1-q_{3}\right)\left(1-q_{4}\right)p_{1/1}+q_{1}\mathrm{Pr}\left\{Q_{2}\neq 0\right\}\left(1-q_{2}\right)q_{3}\left(1-q_{4}\right)p_{1/1,3}\\ \; & +q_{1}\mathrm{Pr}\left\{Q_{2}\neq 0\right\}\left(1-q_{2}\right)\left(1-q_{3}\right)q_{4}p_{1/1,4}+q_{1}\mathrm{Pr}\left\{Q_{2}\neq 0\right\}\left(1-q_{2}\right)q_{3}q_{4}p_{1/1,3,4}\\ \; & +q_{1}\mathrm{Pr}\left\{Q_{2}\neq 0\right\}q_{2}\left(1-q_{3}\right)\left(1-q_{4}\right)p_{1/1,2}+q_{1}\mathrm{Pr}\left\{Q_{2}\neq 0\right\}q_{2}q_{3}\left(1-q_{4}\right)p_{1/1,2,3}\\ \; & +q_{1}\mathrm{Pr}\left\{Q_{2}\neq 0\right\}q_{2}\left(1-q_{3}\right)q_{4}p_{1/1,2,4}+q_{1}\mathrm{Pr}\left\{Q_{2}\neq 0\right\}q_{2}q_{3}q_{4}p_{1/1,2,3,4}\\ \; & =q_{1}\left(1-q_{3}\right)\left(1-q_{4}\right)\left[p_{1/1}-q_{2}\mathrm{Pr}\left\{Q_{2}\neq 0\right\}\left(p_{1/1}-p_{1/1,2}\right)\right]\\ \; & +q_{1}q_{3}\left(1-q_{4}\right)\left[p_{1/1,3}-q_{2}\mathrm{Pr}\left\{Q_{2}\neq 0\right\}\left(p_{1/1,3}-p_{1/1,2,3}\right)\right]\\ \; & +q_{1}q_{4}\left(1-q_{3}\right)\left[p_{1/1,4}-q_{2}\mathrm{Pr}\left\{Q_{2}\neq 0\right\}\left(p_{1/1,4}-p_{1/1,2,4}\right)\right]\\ & +q_{1}q_{3}q_{4}\left[p_{1/1,3,4}-q_{2}\mathrm{Pr}\left\{Q_{2}\neq 0\right\}\left(p_{1/13,4}-p_{1/1,2,3,4}\right)\right] \end{array}$$

(42) $$\begin{array}{cc} \mu_{3} & =\mathrm{Pr}\left\{Q_{1}=0,Q_{2}=0\right\}q_{3}\left(1-q_{4}\right)p_{3/3}+\mathrm{Pr}\left\{Q_{1}=0,Q_{2}=0\right\}q_{3}q_{4}p_{3/3,4}\\ & +\mathrm{Pr}\left\{Q_{1}\neq 0,Q_{2}=0\right\}q_{3}\left(1-q_{1}\right)\left(1-q_{4}\right)p_{3/3}+\mathrm{Pr}\left\{Q_{1}\neq 0,Q_{2}=0\right\}q_{3}q_{4}\left(1-q_{1}\right)p_{3/3,4}\\ \; & +\mathrm{Pr}\left\{Q_{1}\neq 0,Q_{2}=0\right\}q_{1}q_{3}\left(1-q_{4}\right)p_{3/1,3}+\mathrm{Pr}\left\{Q_{1}\neq 0,Q_{2}=0\right\}q_{1}q_{3}q_{4}p_{3/1,3,4}\\ \; & +\mathrm{Pr}\left\{Q_{1}=0,Q_{2}\neq 0\right\}q_{3}\left(1-q_{2}\right)\left(1-q_{4}\right)p_{3/3}+\mathrm{Pr}\left\{Q_{1}=0,Q_{2}\neq 0\right\}q_{3}\left(1-q_{2}\right)q_{4}p_{3/3,4}\\ \; & +\mathrm{Pr}\left\{Q_{1}=0,Q_{2}\neq 0\right\}q_{2}q_{3}\left(1-q_{4}\right)p_{3/2,3}+\mathrm{Pr}\left\{Q_{1}=0,Q_{2}\neq 0\right\}q_{2}q_{3}q_{4}p_{3/2,3,4}\\ \; & +\mathrm{Pr}\left\{Q_{1}\neq 0,Q_{2}\neq 0\right\}q_{3}\left(1-q_{1}\right)\left(1-q_{2}\right)\left(1-q_{4}\right)p_{3/3}\\ \; & +\mathrm{Pr}\left\{Q_{1}\neq 0,Q_{2}\neq 0\right\}q_{2}q_{3}\left(1-q_{1}\right)\left(1-q_{4}\right)p_{3/2,3}\\ \; & +\mathrm{Pr}\left\{Q_{1}\neq 0,Q_{2}\neq 0\right\}q_{3}q_{4}\left(1-q_{1}\right)\left(1-q_{2}\right)p_{3/3,4}\\ \; & +\mathrm{Pr}\left\{Q_{1}\neq 0,Q_{2}\neq 0\right\}q_{1}q_{3}\left(1-q_{2}\right)\left(1-q_{4}\right)p_{3/1,3}\\ \; & +\mathrm{Pr}\left\{Q_{1}\neq 0,Q_{2}\neq 0\right\}q_{2}q_{3}q_{4}\left(1-q_{1}\right)p_{3/2,3,4}\;+\;\mathrm{Pr}\left\{Q_{1}\neq 0,Q_{2}\neq 0\right\}q_{1}q_{2}q_{3}\left(1-q_{4}\right)p_{3/1,2,3}\\ \; & +\mathrm{Pr}\left\{Q_{1}\neq 0,Q_{2}\neq 0\right\}q_{1}q_{3}q_{4}\left(1-q_{2}\right)p_{3/1,3,4}+\mathrm{Pr}\left\{Q_{1}\neq 0,Q_{2}\neq 0\right\}q_{1}q_{2}q_{3}q_{4}p_{3/1,2,3,4} \end{array}$$

Similarly, we can write the service probabilities for $S_{2}$ and $S_{4}$. As we can observe, we see that the service probability of $S_{1}$ depends on the state of the queue of $S_{2}$ and vice versa. Thus, the queues are coupled, which is a known problem and closed form solutions cannot be obtained for more than three users. Furthermore, the service probabilities of $S_{3}$ and $S_{4}$ depend on the joint PDF of the queues of $S_{1}$ and $S_{2}$.

A way to bypass the difficulty due to coupling among the queues is to assume independence as in [13], or if we further assume that the queues have finite capacity then we can use semi-analytical methods from queuing theory. In the first case, we can approximate the performance and that approximation is tight for higher values of the arrival probabilities. After characterizing the service probabilities for each node, we can use the provided analysis in the earlier sections. In a scenario where we have only one user with a queue and *N* users with the generate-at-will policy, the extension becomes a trivial exercise of our analytical results since one can use the expressions directly.

## 6. Conclusions

In this work, we considered a two-user multiple access channel in which both users have AoI-oriented traffic, but with different characteristics. All transmission channels were assumed to be subject to path loss and fading. We have investigated the performance of the average AoI of the first user for the FCFS, LCFS Geo/Geo/1 with preemption, and queue with replacement. Additionally, we have derived the AoI and the average AoI of the second user by considering a threshold for the AoI of the second user. Then, we have formulated an optimization problem to minimize the average AoI of the first user with a constraint on the average AoI of the second user. To solve the proposed optimization problem, we used the interior-point method. Numerical results showed the performance of the proposed algorithm for the different parameters of the system and the impact of multiple antennas.

Future extensions of this work include larger topologies, as discussed in Section 5. Furthermore, an interesting extension is to consider more elaborate schemes at the physical layer such as the MMSE receiver or zero-forcing. Another interesting direction is to consider power control schemes with dynamic programming methodologies such as Markov Decision Processes or stochastic optimization.

## Figures and Tables

**Table 1:** The minimum average AoI of the first user and the optimal values of $\lambda^{*}$, $q_{1}^{*}$, and $q_{2}^{*}$ for $A_{\max}=2$.

γ	$\lambda^{*}$	$q_{1}^{*}$	$q_{2}^{*}$	The Minimum Average AoI of the First User
−5 dB	0.6159	1	0.6047	3.10
−3 dB	0.5264	1	0.6443	3.54
−1 dB	0.4263	1	0.6859	4.20
1 dB	0.3264	1	0.7175	5.16
3 dB	0.2425	1	0.7289	6.46
5 dB	0.1832	1	0.7239	8.02

**Table 2:** The minimum average AoI of the first user and the optimal values of $\lambda^{*}$, $q_{1}^{*}$, and $q_{2}^{*}$ for $A_{\max}=5$.

γ	$\lambda^{*}$	$q_{1}^{*}$	$q_{2}^{*}$	The Minimum Average AoI of the First User
−5 dB	0.7539	1	0.2477	2.59
−3 dB	0.6947	1	0.2684	2.78
−1 dB	0.6260	1	0.2935	3.02
1 dB	0.5519	1	0.3196	3.32
3 dB	0.4806	1	0.3416	3.67
5 dB	0.4208	1	0.3560	4.04

**Table 3:** The minimum average AoI of the first user and the optimal values of $\lambda^{*}$, $q_{1}^{*}$, and $q_{2}^{*}$ for $A_{\max}=10$.

γ	$\lambda^{*}$	$q_{1}^{*}$	$q_{2}^{*}$	The Minimum Average AoI of the First User
−5 dB	0.8246	1	0.1257	2.39
−3 dB	0.7815	1	0.1376	2.50
−1 dB	0.7303	1	0.1531	2.64
1 dB	0.6730	1	0.1708	2.81
3 dB	0.6147	1	0.1880	3.01
5 dB	0.5622	1	0.2019	3.19

**Table 4:** The minimum average AoI of the first user and the optimal values of $\lambda^{*}$, $q_{1}^{*}$, and $q_{2}^{*}$ for $A_{\max}=15$.

γ	$\lambda^{*}$	$q_{1}^{*}$	$q_{2}^{*}$	The Minimum Average AoI of the First User
−5 dB	0.8563	1	0.0844	2.31
−3 dB	0.8206	1	0.0929	2.40
−1 dB	0.7777	1	0.1043	2.51
1 dB	0.7288	1	0.1179	2.63
3 dB	0.6775	1	0.1320	2.78
5 dB	0.6297	1	0.1441	2.92

## Data Availability

Not applicable.

## Appendix A

To prove the convexity of the objective function, we first define the expression given in (38a) as

(A1) $$\begin{array}{c} A=\frac{1}{\lambda }+\frac{1-\lambda }{p_{1/1}-q_{2}\left(p_{1/1}-p_{1/1,2}\right)-\lambda }-\frac{\lambda \left(1-p_{1/1}+q_{2}\left(p_{1/1}-p_{1/1,2}\right)\right)}{{\left(p_{1/1}-q_{2}\left(p_{1/1}-p_{1/1,2}\right)\right)}^{2}}. \end{array}$$

Now, by taking the second derivative $\frac{d^{2}A}{d\lambda^{2}}$ we have

(A2) $$\begin{array}{c} \frac{d^{2}A}{d\lambda^{2}}=\frac{2}{\lambda^{3}}+\frac{2(1-X)}{{\left(X-\lambda \right)}^{3}} \end{array}$$

where $X=p_{1/1}-q_{2}\left(p_{1/1}-p_{1/1,2}\right)$, and using the expression given in (38c), we have λ<X⩽1. Therefore, for all values of *X* and λ, the second derivative $\frac{d^{2}A}{d\lambda^{2}}$ is positive, and thus the objective function is a convex function of λ when $q_{2}$ is fixed. Similarly, by taking the second derivative $\frac{d^{2}A}{dq_{2}^{2}}$ when λ is fixed we have

(A3) $$\begin{array}{c} \frac{d^{2}A}{dq_{2}^{2}}={\left(p_{1/1}-p_{1/1,2}\right)}^{2}\left[\frac{-2\lambda (3-X)}{X^{4}}+\frac{2(1-\lambda )}{{\left(X-\lambda \right)}^{3}}\right] \end{array}$$

where $X=p_{1/1}-q_{2}\left(p_{1/1}-p_{1/1,2}\right)$ and λ<X⩽1. It can be readily shown that for all values of *X* and λ, $\frac{d^{2}A}{dq_{2}^{2}}$ is positive and therefore the objective function A is a convex function of $q_{2}$ when λ is fixed.

## Appendix B

In order to prove the convexity of the objective function given in (40a), we must verify that the Hessian matrix of the objective function is positive semi-definite. We first consider the objective function as

(A4) $$\begin{array}{c} B=\frac{1}{\lambda }+\frac{1}{p_{1/1}-q_{2}\left(p_{1/1}-p_{1/1,2}\right)}. \end{array}$$

To obtain the Hessian matrix, we need to derive $\frac{d^{2}B}{d\lambda^{2}}$, $\frac{d^{2}B}{d\lambda dq_{2}}$, and $\frac{d^{2}B}{dq_{2}^{2}}$.

(A5) $$\begin{array}{cc} \frac{d^{2}B}{d\lambda^{2}} & =\frac{2}{\lambda^{3}}⩾0\\ \frac{d^{2}B}{d\lambda dq_{2}} & =0\\ \frac{d^{2}B}{dq_{2}^{2}} & =\frac{2(p_{1/1}-p_{1/1,2})}{{\left(p_{1/1}-q_{2}\left(p_{1/1}-p_{1/1,2}\right)\right)}^{3}}⩾0. \end{array}$$

According to the stability constraint in (40c), $\lambda <p_{1/1}-q_{2}\left(p_{1/1}-p_{1/1,2}\right)⩽1$ and therefore $\frac{d^{2}B}{dq_{2}^{2}}⩾0$. Since $\frac{d^{2}B}{d\lambda^{2}}\times \frac{d^{2}B}{dq_{2}^{2}}-{\left(\frac{d^{2}B}{d\lambda dq_{2}}\right)}^{2}>0$, the Hessian matrix is positive semi-definite and the objective function is convex.
